# AI-based early mental health screening for local public officials

**DOI:** 10.3389/fpubh.2026.1724307

**Published:** 2026-03-20

**Authors:** Sangwon Lee, Bokyung Seo, Sangkook Kim, Jungae Park, Jingyeong An, Jeongsook Kim, Young-A Ji

**Affiliations:** 1 Incheon City Hall, Incheon, Republic of Korea; 2 Gyeonggi Provincial Government, Suwon, Republic of Korea; 3 Hwaseong City Hall, Hwaseong, Republic of Korea; 4 Ansan City Hall, Ansan, Republic of Korea; 5 Hanam City Hall, Hanam, Republic of Korea; 6 Gunpo City Hall, Gunpo, Republic of Korea; 7 Department of Medical Education, School of Medicine, Sungkyunkwan University, Seoul, Republic of Korea

**Keywords:** AI-based mental health, burnout, early diagnosis, public officials, stigma

## Abstract

**Introduction:**

Local public officials are consistently exposed to high levels of occupational stress and burnout due to heavy workloads, citizen interactions, and rigid organizational cultures. Conventional self-report assessments are limited by subjectivity and stigma, whereas artificial intelligence (AI)-based screening systems offer potential advantages for earlier engagement and improved accessibility, particularly in stigma-sensitive contexts. This study evaluated the acceptability and perceived usefulness of an AI-based mental health screening system for local officials.

**Methods:**

A two-stage survey was conducted with 126 officials in local public. Stage one employed standardized instruments (PHQ-9, GAD-7, job stress, stigma perception). Stage two included 30 randomly selected participants who completed both self-report and GPT-based AI assessments, followed by evaluation of satisfaction and acceptance. Analyses included descriptive statistics, ANOVA, Chi-square tests, reliability (Cronbach’s *α*), correlation, and multiple regression using SPSS 29.0.

**Results:**

Respondents reported high job stress, with younger officials showing greater vulnerability. Despite only 3.2% having prior AI diagnostic experience, 73.8% trusted AI results more than self-reports, and 84.9% expressed willingness for regular participation. Stigma toward conventional services remained prevalent, yet 81.7% agreed AI could mitigate stigma. In the in-depth survey, AI screening was rated highly for usability, efficiency, interpretability, and re-engagement intention, although some concerns regarding transparency and interpretability remained. Therefore, the AI model in this study should not be interpreted as providing clinical diagnoses. Its outputs reflect pattern recognition within conversational data, and further validation against clinician-administered psychiatric assessments is required. Regression analysis showed that trust in AI results (*β* = 0.463, *p* < 0.001) and perceived stigma reduction (*β* = 0.432, *p* < 0.001) significantly predicted AI acceptance.

**Conclusion:**

AI-based screening demonstrates strong potential as a perceived potential and stigma-free tool for public officials. To ensure sustainable implementation, institutional safeguards and algorithm refinement are required.

## 1 Introduction

Local public officials are consistently exposed to substantial occupational stress and psychological burden arising from heavy workloads, frequent civil complaints, insufficient manpower, hierarchical organizational culture, and extended working hours (1, 2). Such working conditions have been shown to contribute to a wide spectrum of mental health problems, including depression, anxiety, and burnout. For example, job demands, inadequate compensation, job insecurity, and supervisor control have been identified as major predictors of depressive symptoms among local officials, demonstrating the structural nature of their mental health risks (3).

National data further illustrate the magnitude of this issue. According to the 2022 compensation statistics for public officials, the number receiving medical treatment for mental illness was 2.14 per 10,000—approximately 11 times higher than the rate among general workers (0.19 per 10,000). Deaths due to suicide or other mental illnesses were also markedly higher in this population (0.17 vs. 0.02 per 10,000), underscoring that mental health concerns within the public sector constitute not only an individual health problem but also a broader structural and societal challenge (4).

Despite these risks, many public officials do not receive timely or adequate support. One survey found that 28.1% of officials reported severe stress, yet 61.1% did not take any action despite experiencing symptoms; additionally, nearly half were unaware of available community mental health services (5). These findings suggest critical gaps in the current institutional support system.

Mental health assessment in the public sector still relies primarily on self-report questionnaires such as the PHQ-9 and GAD-7, supplemented by face-to-face counseling (6). However, these approaches depend heavily on respondents’ insight and willingness to disclose symptoms, which can be hindered by stigma, fear of negative evaluation, and concerns about confidentiality (7). Indeed, 73.6% of individuals in the general population with mental health problems have not received professional support (8), and such barriers may be even more pronounced within public organizations due to worries about how diagnostic information might be used. Consequently, existing approaches remain limited in their ability to facilitate earlier engagement with screening resources and meaningful engagement.

In response to these limitations, AI-based early screening systems have gained increasing attention. AI can analyze unstructured data—including conversational patterns, speech, keystroke dynamics, and biometric signals—to infer users’ emotional states in real time (9, 10). Large language models (LLMs), in particular, can detect linguistic markers indicative of depression, anxiety, and stress by examining semantic patterns and contextual cues. GPT-based models have demonstrated approximately 85% concordance with expert judgments when mapping conversational expressions to PHQ-9 domains (11). Similarly, the AI-driven chatbot “Dr. Listen” showed 84% accuracy in identifying high-risk groups, along with high user acceptance (12).

Compared with traditional self-report questionnaires, AI-based systems offer several advantages: reduced susceptibility to response bias, multidimensional and continuous monitoring capability, and the potential to track dynamic emotional changes over time. When predetermined thresholds are met, these systems can automatically recommend counseling or alert relevant services (13). Importantly, anonymity and the non-face-to-face nature of AI interactions may mitigate stigma and enhance accessibility, thereby strengthening preventive mental-health management systems for public officials (10).

Most importantly, public-sector mental health should be understood not only as an individual-level psychological issue but as an outcome shaped by structural working conditions including workload, emotional labor, limited autonomy, and hierarchical culture. Therefore, the potential value of AI-based screening lies not in replacing existing systems or shifting responsibility to individuals, but in supplementing organizational mental-health governance by reducing stigma, increasing access, and enabling earlier detection within institutional constraints. Consistent with organizational stress models such as the Job Demand-Control-Support framework, AI-based screening tools in this study are conceptualized as supporting components embedded within a broader institutional ecosystem rather than as isolated technological solutions (14).

Consistent with organizational stress models such as the Job Demand-Control-Support framework, mental health among public officials is shaped primarily by structural conditions workload, emotional labor, limited job control, and hierarchical culture rather than by individual vulnerabilities. Accordingly, AI-based screening tools should be understood as complementary supports within broader organizational mental-health ecosystems, not as substitutes for systemic policy reforms or professional care.

Against this backdrop, the present study examines the acceptability and perceived utility of an AI-based mental health screening system among local public officials, with particular attention to trust, stigma-related perceptions, and user experience. Rather than evaluating diagnostic accuracy or detection performance, the study focuses on how AI-based screening is perceived as a potential supplementary tool within organizational mental-health contexts. The following hypotheses guided the investigation:

*Hypothesis 1*. Higher levels of occupational stress among public officials will be associated with an increased perceived need for mental health management.

*Hypothesis 2*. Compared with face-to-face counseling, AI-based self-screening will be associated with lower perceived stigma, thereby enhancing access to mental health services.

## 2 Materials and methods

### 2.1 Study design

This study sought to empirically perception of an AI-based early mental health screening system for local public officials. In particular, it compared the AI-based diagnostic approach with conventional self-report measures to examine whether AI could more effectively detect high-risk individuals. Furthermore, the study investigated the mediating and moderating roles of occupational stress and perceived stigma in influencing mental health outcomes.

First, the study assessed the mental health status of local officials and identified high-risk groups according to occupational characteristics. To this end, a structured survey was administered, incorporating indicators such as emotional state, work-related fatigue, sleep quality, and job engagement over the past 6 months.

Second, the study evaluated the extent to which public officials accepted the AI-based screening system and the benefits they expected from its potential implementation. Given its flexibility in terms of time and location, AI screening allows for repeated self-assessment and longitudinal tracking of mental health changes. The study therefore examined awareness of these advantages and assessed perceived feasibility and validity of the system in real-world settings.

Third, the study analyzed the role of perceived stigma related to mental health services and explored the potential of AI screening to mitigate such barriers. Since workplace stigma often prevents public officials from seeking counseling or diagnostic services, perceived stigma levels were measured, and the ability of AI-based screening to reduce these psychological barriers was empirically tested.

### 2.2 Participants and procedure

All 412 officials in Gyeonggi Province were invited via institutional email, and 126 participated (response rate: 30.6%). A convenience volunteer sampling approach was used. All participants completed the Stage 1 general survey, and a randomly selected subsample of 30 officials subsequently participated in the Stage 2 in-depth survey.

The procedure consisted of two stages: Stage 1 (General Survey): Participants completed self-report questionnaires assessing mental health status (PHQ-9, GAD-7), occupational stress, and perceived stigma toward mental health services. Stage 2 (In-depth Survey): A subsample of Stage 1 respondents underwent both self-report assessments and an AI-based diagnostic evaluation using a GPT-driven system. They also completed additional items measuring acceptance and satisfaction with the diagnostic method.

To confirm the adequacy of the sample size, *a priori* power analysis was conducted using G*Power. Results indicated that the sample of 126 participants provided more than 80% statistical power for detecting a medium effect size (*f* = 0.25) in ANOVA with four groups at *α* = 0.05, thereby ensuring the statistical validity of the sample.

### 2.3 Measures and analytical plan

Mental health status was assessed using standardized self-report instruments: the Patient Health Questionnaire-9 (PHQ-9) and the Generalized Anxiety Disorder-7 (GAD-7). For AI-based assessment, a GPT-driven diagnostic model was applied to evaluate participants’ mental health status through natural language inputs.

The primary variables of interest included acceptance of AI-based diagnosis, classification of high-risk mental health status, occupational stress, perceived stigma toward mental health services, and preferences between diagnostic modalities.

All statistical analyses were conducted using SPSS Statistics version 29.0 (IBM Corp., Armonk, NY, United States). Descriptive statistics and frequency analyses were performed to summarize sample characteristics. Internal consistency reliability of the scales was assessed using Cronbach’s *α*. Pearson’s correlation analyses were employed to examine associations among key variables. Multiple regression analyses were conducted to identify predictors of AI acceptance, with occupational stress and perceived stigma as independent variables. Group differences were tested using analysis of variance (ANOVA) for continuous outcomes and Chi-square (*χ*^2^) tests for categorical outcomes. Table 1 summarizes the overall research procedures.

**Table 1:** Methods and procedure.

Category	Content
Study population	Local government officials (stage 1: *n* = 126, stage 2: *n* = 30)
Step 1 general survey	Self-reported questionnaire (PHQ-9, GAD-7, job stress, perceived stigma)
Step 2 in-survey	Comparison of self-report measures and AI-based screening experiences
Comparison metrics	Perceived credibility, usability, acceptance, and perceived stigma reduction
Analytical tools	SPSS Statistics 29.0; reliability (Cronbach’s *α*), ANOVA, *χ*^2^, regression, correlation analysis
Research objective	To examine the acceptability of AI-based screening methods

It should be noted that the AI-based screening used in this study does not constitute a validated clinical diagnostic system. Rather, it provides conversational, model-driven outputs whose perceived usefulness and acceptability were evaluated. No claims regarding clinical accuracy or diagnostic concordance with mental-health professionals are made.

### 2.4 Description of the AI-based screening system

The AI-based screening system used in this study is a GPT-based LLM that employs natural language processing to analyze short, text-based user responses. The AI interaction was fully standardized and identical for all participants. Each participant responded to the same fixed set of structured conversational prompts designed to reflect emotional domains commonly assessed by the PHQ-9 and GAD-7, rather than engaging in free, adaptive, or personalized dialog. The AI system therefore functioned as a standardized stimulus to elicit participants’ perceptions of credibility, usability, and acceptability. It was not intended to operate as a diagnostic or evaluative instrument, nor to assess diagnostic accuracy or clinical performance. The model was not newly trained or fine-tuned for the purposes of this study; instead, it relied on a pre-trained LLM developed using large-scale linguistic datasets. While previous studies have reported approximately 80–85% concordance between similar GPT-based systems and clinician-interpreted emotional indicators, no such validation or concordance testing was conducted within the present sample. The system generated simple categorical output labels intended solely to support user reflection and engagement, rather than to provide clinical diagnoses, quantitative risk scores, or decision thresholds. Because the AI interaction was standardized, non-diagnostic, and identical across participants, responses to the system were interpreted as evaluations of perceived credibility, usability, trustworthiness, and overall acceptability, rather than as indicators of diagnostic performance.

## 3 Results

### 3.1 Stage 1: general survey results

#### 3.1.1 General characteristics of participants

A total of 126 local government officials participated in the Stage 1 survey. Among them, 36.5% were male (*n* = 46) and 63.5% were female (*n* = 80). The age distribution indicated that 1.6% were in their 20s (*n* = 2), 10.3% in their 30s (*n* = 13), 50.8% in their 40s (*n* = 64), and 37.3% were aged 50 years or older (*n* = 47) (see Table 2). Regarding years of service, the majority (86.5%, *n* = 109) had ≥10 years of experience, followed by 8.7% (*n* = 11) with 5–10 years, and 4.8% (*n* = 6) with <5 years. Occupational categories were diverse, with administrative positions accounting for 51.6% (*n* = 65), followed by technical positions 20.6% (*n* = 26), social welfare/health positions 13.5% (*n* = 17), and others 14.3% (*n* = 18) in Table 1. This distribution suggests that the sample was not skewed toward a particular subgroup, reflecting a broad cross-section of local government officials in Table 3.

**Table 2:** Demographic characteristics of participants (*n* = 126).

Variable	Category	*n*	%
Gender	Male	46	36.5
	Female	80	63.5
Age group	20s	2	1.6
	30s	13	10.3
	40s	64	50.8
	50s and above	47	37.3
Years of service	<5 years	6	4.8
	5–10 years	11	8.7
	≥10 years	109	86.5
Job type	Administrative	65	51.6
	Engineering/technical	26	20.6
	Social welfare/health	17	13.5
	Others	18	14.3

**Table 3:** General characteristics of participants (*n* = 126).

Characteristic	Category	*n*	%
Sex	Male	46	36.5
	Female	80	63.5
Age	20s	2	1.6
	30s	13	10.3
	40s	64	50.8
	≥50 years	47	37.3
Years of service	<5 years	6	4.8
	5–10 years	11	8.7
	≥10 years	109	86.5
Occupation	Administrative	65	51.6
	Technical	26	20.6
	Social welfare/health	17	13.5
	Others	18	14.3

#### 3.1.2 Job stress and perceived organizational support

A total of 58.7% of respondents reported experiencing severe job-related stress within the past 6 months. More than half also indicated significant fatigue due to frequent civil complaints and task changes. The mean burnout index was 3.7 points, reflecting a moderate-to-high level of occupational stress.

Group comparisons showed a statistically significant difference in burnout scores by age (ANOVA, *F* = 3.43, *p* = 0.019), with officials in their 20s–30s reporting higher stress levels than those aged 40 years or older. No statistically significant differences were observed by years of service (*F* = 2.44, *p* = 0.091) or sex (*F* = 0.10, *p* = 0.753). These results suggest that younger and early-career officials constitute a particularly vulnerable group in terms of mental health in Table 4.

**Table 4:** Differences in burnout index by demographic characteristics.

Variable	*F*-value	*p*-value
Age group	*F* = 3.43	0.019*
Years of service	*F* = 2.44	0.091
Sex	*F* = 0.10	0.753

*p < 0.05.

#### 3.1.3 Acceptance and trust in AI-based diagnosis

Overall, acceptance of AI-based mental health diagnosis was high. A total of 70.6% of respondents agreed that AI screening would be helpful for mental health management, and 84.9% expressed willingness to participate if regular self-assessment opportunities were provided. Although only 3.2% had prior experience with AI-based diagnosis, 73.8% (*n* = 93) reported trusting AI results more than conventional self-report questionnaires, compared to 26.2% (*n* = 33) who preferred self-reports.

By subgroup, trust in AI was observed in 100% of respondents in their 20s, 76.9% in their 30s, 73.4% in their 40s, and 72.3% among those aged 50 and older. Similar patterns were found for years of service, with 100% trust among those with <5 years, 63.6% among those with 5–10 years, and 73.4% among those with ≥10 years. Both men (76.1%) and women (72.5%) showed higher trust in AI than in self-reports. Chi-square tests indicated no statistically significant differences across age (*χ*^2^ = 0.83, *p* = 0.842), years of service (*χ*^2^ = 2.73, *p* = 0.256), or sex (*χ*^2^ = 0.05, *p* = 0.818), suggesting that acceptance of AI-based diagnosis was consistently high across demographic subgroups in Table 5.

**Table 5:** Differences in trust toward AI-based vs. self-report diagnosis by demographic characteristics.

Variable	Category	Self-report *n* (%)	AI *n* (%)	*χ* ^2^	*p*
Total		33 (26.2%)	93 (73.8%)		
Age group	20s	0 (0.0%)	2 (100.0%)	*χ*^2^ = 0.83	0.842
	30s	3 (23.1%)	10 (76.9%)		
	40s	17 (26.6%)	47 (73.4%)		
	50s+	13 (27.7%)	34 (72.3%)		
Years of service	<5 years	0 (0.0%)	6 (100.0%)	*χ*^2^ = 2.73	0.256
	5–10 years	4 (36.4%)	7 (63.6%)		
	≥10 years	29 (26.6%)	80 (73.4%)		
Sex	Male	11 (23.9%)	35 (76.1%)	*χ*^2^ = 0.05	0.818
	Female	22 (27.5%)	58 (72.5%)		

#### 3.1.4 Perceived stigma toward mental health services

A total of 54.8% of respondents agreed that seeking mental health counseling could result in negative peer evaluations, and nearly half expressed concerns about potential disadvantages in personnel evaluations or work assignments. By contrast, 81.7% agreed that AI-based self-screening could help alleviate such stigma. The mean stigma index score was 3.3, with no significant differences across demographic variables (age, sex, years of service).

#### 3.1.5 Reliability and correlation analysis

The internal consistency of the scales was verified using Cronbach’s *α*. Both the AI acceptance scale (*α* = 0.838) and the stigma perception scale (*α* = 0.868) demonstrated high reliability, while the job stress scale showed lower reliability (*α* = 0.660), which may be attributed to its two-item structure.

Correlation analysis revealed a moderate positive association between AI acceptance and the belief that AI-based diagnosis can reduce stigma (*r* = 0.533, *p* < 0.01), as well as with trust in AI results (*r* = 0.412, *p* < 0.01). Conversely, correlations with job stress (r = 0.133) and general stigma perception (*r* = 0.255) were weak and non-significant. A significant positive correlation was also observed between general stigma perception and the belief that AI reduces stigma (*r* = 0.424, *p* < 0.01), suggesting that those more aware of stigma were also more likely to view AI as a mitigating factor in Table 6.

**Table 6:** Reliability and correlation analysis of study variables.

Variable	Items	Cronbach’s *α*	1	2	3	4	5
1. AI acceptance	4	0.838	1				
2. Stigma perception	4	0.868	0.255	1			
3. Job stress	2	0.660	0.133	0.271	1		
4. Trust in AI results	1	N/A	0.412**	0.132	0.154	1	
5. AI reduces stigma	1	N/A	0.533**	0.424**	0.147	0.147	1

**p < 0.01.

#### 3.1.6 Regression analysis of predictors of AI acceptance

To examine predictors of AI acceptance, a multiple regression analysis was conducted. The overall model was statistically significant, *R*^2^ = 0.398, *F* (4,121) = 19.97, *p* < 0.001, indicating that approximately 39.8% of the variance in AI acceptance was explained by the included predictors.

Among the independent variables, trust in AI results (*β* = 0.463, *p* < 0.001) and the belief that AI can reduce stigma (*β* = 0.432, *p* < 0.001) were significant positive predictors of AI acceptance. By contrast, job stress (*β* = 0.006, *p* = 0.907) and general stigma perception (*β* = 0.004, *p* = 0.948) did not significantly predict AI acceptance.

These findings suggest that AI acceptance is not directly influenced by baseline job stress or general stigma levels but is instead more strongly shaped by confidence in AI’s diagnostic credibility and the expectation that AI can alleviate stigma in Table 7.

**Table 7:** Multiple regression analysis predicting AI acceptance.

Independent variable	*B*	SE	*β*	*t*	*p*
Constant	1.875	0.298	—	6.297	<0.001
Job stress	0.006	0.049	0.006	0.117	0.907
Perceived stigma	0.004	0.063	0.004	0.065	0.948
Trust in AI results	0.463	0.098	0.463	4.710	<0.001
Perceived stigma reduction by AI	0.432	0.071	0.432	6.118	<0.001

Model fit: *R*^2^ = 0.398, Adj. *R*^2^ = 0.378, *F* (4,121) = 19.97, *p* < 0.001.

### 3.2 Stage 2 in-depth survey results

A follow-up survey was conducted with 30 randomly selected participants who completed the AI-based psychological assessment. Comprehension of the test items (*M* = 4.13, SD = 0.73) and convenience of the process (*M* = 4.10, SD = 0.84) were rated highly. The perception that the results accurately reflected participants’ current psychological state was somewhat lower (*M* = 3.77, SD = 0.94), but trust in the AI-based results remained high (*M* = 4.07, SD = 0.78). Compared with traditional assessments, 80.0% of participants reported that the AI-based test was more accurate or helpful (*M* = 4.13, SD = 0.86). Ease of interpreting the results (*M* = 4.17, SD = 0.83) and efficiency of the overall process (*M* = 4.20, SD = 0.85) were also positively evaluated. Notably, willingness to participate again received the highest score (*M* = 4.27, SD = 0.83), with 83.3% of participants expressing strong intention to re-engage in future AI-based assessments.

## 4 Discussion

This study examined the acceptability of an AI-based mental health screening system among local public officials, focusing on perceptions of stigma, diagnostic trust, and satisfaction with the testing process. Consistent with prior research, the findings confirm that public officials particularly younger and early-career employees experience substantial occupational stress and burnout (15). These levels of psychosocial strain reflect well-documented characteristics of public-sector work environments, including heavy citizen-facing responsibilities, limited autonomy, and hierarchical administrative structures.

To situate these findings conceptually, the results can be interpreted within the Job Demand–Control–Support model and the Technology Acceptance Model (TAM). TAM posits that perceived usefulness and trust strongly influence acceptance of new technologies, which aligns with the present study’s regression results showing that trust in AI output and perceived stigma reduction were the strongest predictors of AI acceptance. At the same time, organizational stress theories emphasize that psychological burden is shaped not by individual deficiencies but by structural conditions. This reinforces that AI-based screening cannot replace systemic reforms but may serve as a complementary mechanism within broader organizational mental-health governance.

The high acceptability of AI-based screening observed in this study regardless of prior experience with AI—suggests strong user receptivity. Participants reported greater trust in AI-based assessments than conventional self-report measures, suggesting concerns about subjectivity, social desirability bias, or potential organizational consequences associated with face-to-face disclosure (16). Notably, acceptance levels were consistent across demographic subgroups, suggesting that AI-based screening was broadly acceptable within this specific public-sector context, without substantial demographic disparities. However, given the exploratory design and sampling limitations, these findings should be interpreted as context-specific rather than universally generalizable.

Stigma emerged as a significant barrier to accessing mental health services, echoing previous findings that public officials often avoid counseling due to fears of negative evaluation or workplace repercussions (17). In this context, the non-face-to-face and de-identified nature of AI-based screening was perceived as a meaningful advantage. Participants believed that AI-based assessments could reduce stigma by enabling more private, autonomous, and judgment-free engagement with mental-health screening tools.

Beyond individual-level perceptions, the findings offer insights into how AI-based screening may function within organizational systems. Importantly, the significant association between AI acceptance and the belief that “AI can reduce stigma” suggests that employees view AI as a structural support mechanism rather than a replacement for professional care. For organizational implementation, this implies that acceptance is contingent on transparency, institutional safeguards, and assurances that AI-generated information will not be misused in personnel decisions (18).

From an academic perspective, this study contributes early empirical evidence on AI-enabled mental health screening for public-sector employees—a group under-examined in prior digital mental-health research, which has largely focused on university students or general workers (19). By incorporating occupational context and governance considerations, the findings extend existing literature on digital health adoption and organizational mental-health culture.

Practically, the study highlights several policy implications. First, mental-health systems should leverage the stigma-reducing advantages of anonymous, technology-assisted screening to extend early detection and support. Second, institutional safeguards must clearly delineate boundaries between mental-health management and performance evaluation to prevent psychological data from being misused. Third, efforts should be made to strengthen the validity, transparency, and interpretability of AI-based outputs to enhance user trust. Fourth, targeted support programs are needed for early-career officials, who exhibited higher stress levels.

Beyond these implications, ethical and governance issues require careful consideration. Although this study collected only anonymized textual input and stored no identifiable conversational data, AI systems inherently pose potential risks, including algorithmic bias stemming from pre-trained datasets, cultural misalignment, and the possibility of secondary misuse if data-governance structures are weak. As such, AI-based tools must be implemented within robust institutional frameworks that ensure privacy protection, algorithmic transparency, and clear restrictions on organizational use of the data.

Finally, it is essential to avoid inadvertently promoting an individual-responsibility narrative. While AI-based tools can facilitate early identification and reduce stigma, they must operate within comprehensive organizational mental-health strategies that address workload, staffing adequacy, and structural support. Viewing AI as a supplementary rather than replacement mechanism is therefore crucial.

This study also has limitations. The sample size limits generalizability, particularly for subgroup analysis. The cross-sectional design precludes conclusions regarding temporal or causal relationships. Additionally, the smaller sample in the in-depth survey renders those findings exploratory. Future research should include larger and more diverse samples across regions, employ longitudinal designs to evaluate long-term mental-health trajectories, and conduct validation studies comparing AI-based assessments with clinical diagnostic evaluations (20).

## 5 Conclusion

This study examined the acceptability of an AI-based mental health screening approach, perceptions of stigma, and satisfaction among local public officials with direct test experience. The findings revealed that public officials were exposed to high levels of occupational stress, and that AI-based screening, regardless of prior experience, were broadly trusted and accepted. Moreover, the non-face-to-face and de-identified nature of AI assessments demonstrated potential in reducing stigma associated with accessing mental health services.

These results suggest that AI-based diagnostic systems can serve as an innovative tool for mental health management in the public sector. For successful implementation, several conditions are essential: institutionalization of regular self-screening, safeguards to prevent any linkage with performance evaluations, refinement of algorithms and interpretive frameworks to enhance perceived transparency, interpretability, and user trust, while future studies are required to establish diagnostic validity, and the strengthening of tailored support programs for vulnerable groups experiencing higher stress.

This study holds both academic and practical significance as an initial empirical investigation into the institutional feasibility and limitations of AI-based mental health management among public officials. Future research should employ larger and more diverse samples with longitudinal designs to validate these findings and conduct cross-validation studies with clinical diagnoses to further strengthen the reliability and utility of AI-based assessments.

## Data Availability

The raw data supporting the conclusions of this article will be made available by the authors, without undue reservation.
